# Immune cells in diabetic retinopathy: A Mendelian randomization study

**DOI:** 10.1097/MD.0000000000044549

**Published:** 2025-10-10

**Authors:** Pengfei Jiang, Donghua Liu, Yunfeng Yu, Jingyi Wu, Gang Hu, Xinyu Yang, Pei Liu

**Affiliations:** a Quzhou Hospital of Zhejiang Medical and Health Group, Quzhou, Zhejiang, China; b The First Hospital of Hunan University of Chinese Medicine, Changsha, Hunan, China; c The Third School of Clinical Medicine, Zhejiang Chinese Medical University, Hangzhou, Zhejiang, China; d School of Traditional Chinese Medicine, Hunan University of Chinese Medicine, Changsha, Hunan, China.

**Keywords:** diabetic retinopathy, genome-wide association studies, immune cells, Mendelian randomization, phenotype

## Abstract

The role of immune cells in diabetic retinopathy (DR) is unclear. This study aims to assess the causal effect of various immune cells on DR by Mendelian randomization (MR). Immune cell datasets were acquired from European Bioinformatics Institute, and a DR dataset was acquired in FinnGen. Single nucleotide polymorphisms were screened stepwise according to the assumptions of association, independence, and exclusivity. Inverse variance weighted was used as the main method for MR analysis. MR-Egger was used to assess the horizontal pleiotropy of the results. Cochran *Q* and leave-one-out methods were respectively used for heterogeneity and sensitivity of the results. MR analysis identified 11 immune cell phenotypes associated with an increased genetic susceptibility to DR: T cell related phenotypes included CD4 on resting Treg, CD4/CD8br, CD28+ CD45RA− CD8dim AC, and CD28− CD25++ CD8br %T cell; NKT cell related phenotypes included CD8br NKT AC and CD3 on NKT; Dendritic cell related phenotypes included CD80 on plasmacytoid DC, CD62L+ plasmacytoid DC, and myeloid DC; The monocyte related phenotype was HLA DR on CD33br HLA DR+ CD14−; and the myeloid cell related phenotype was CD33− HLA DR− AC. No horizontal pleiotropy was observed (*P* ≥ .05). Cochran *Q* showed no heterogeneity in the results except for CD8br NKT AC (*P* < .001) and HLA DR on CD33br HLA DR+ CD14− (*P* = .002). Sensitivity analysis showed the results were robust. The MR analysis revealed 11 immune cell phenotypes associated with an increased genetic susceptibility to DR. These findings provide a new perspective on the pathogenesis and drug development of DR.

## 1. Introduction

Diabetic retinopathy (DR), a microangiopathy of the retina caused by chronic, persistent hyperglycemia,^[1]^ stands as the primary cause of new vision loss globally.^[2]^ Epidemiological studies indicate that around 451 million people worldwide had diabetes in 2017, a number projected to increase to 693 million by 2045.^[3]^ As the prevalence of diabetes increases worldwide, so does the number of people suffering from diabetes complications.^[4]^ The global prevalence of DR has been reported to be as high as 22.27% among diabetic patients.^[5]^ The main manifestations of DR include blurred vision, eye floaters and distorted vision, and even partial or complete blindness,^[6]^ profoundly impact the quality of life for affected individuals. Although treatments such as anti-vascular endothelial growth factor, laser photocoagulation, and intravitreal steroids have provided some relief from DR, their efficacy remains limited.^[7]^ Therefore, it is crucial to gain a deeper understanding of DR’s pathogenesis to explore potential targets and drugs.

Previous studies have shown that factors such as oxidative stress, amino acid metabolism, and glycated hemoglobin levels are associated with DR.^[8]^ With advancing research, the role of inflammation and immune response in DR’s pathogenesis has gained increasing attention.^[9]^ A previous study has revealed phenotypic changes of certain immune cells in patients with DR, such as significantly higher levels of sCD14s compared to the general population.^[10]^ However, most current studies focus on specific immune cells and lack a comprehensive explanation of diverse immune cells. Additionally, they are predominantly cross-sectional studies and cannot effectively assess the causal attributes between immune cells and DR. Therefore, novel and effective methods are needed to evaluate the causal effects of different immune cells on DR, providing insights for exploring specific immune markers and developing immune-targeted drugs.

The application of analytical techniques such as Mendelian randomization (MR), meta-analysis, bibliometrics, and network pharmacology in medical research is becoming increasingly widespread.^[11–16]^ As a genetic epidemiological method, MR assesses the causal effect of exposure on outcomes using genetic variants as a tool.^[17]^ Compared to traditional research methods, it is less susceptible to reverse causation, selective bias, and confounding factors.^[18]^ In this study, we used MR to analyze the role of 731 immune cell phenotypes on the genetic susceptibility to DR, aiming to reveal the immune cells associated with the onset or progression of DR.

## 2. Materials and methods

### 2.1. Study design

**Figure 1.**
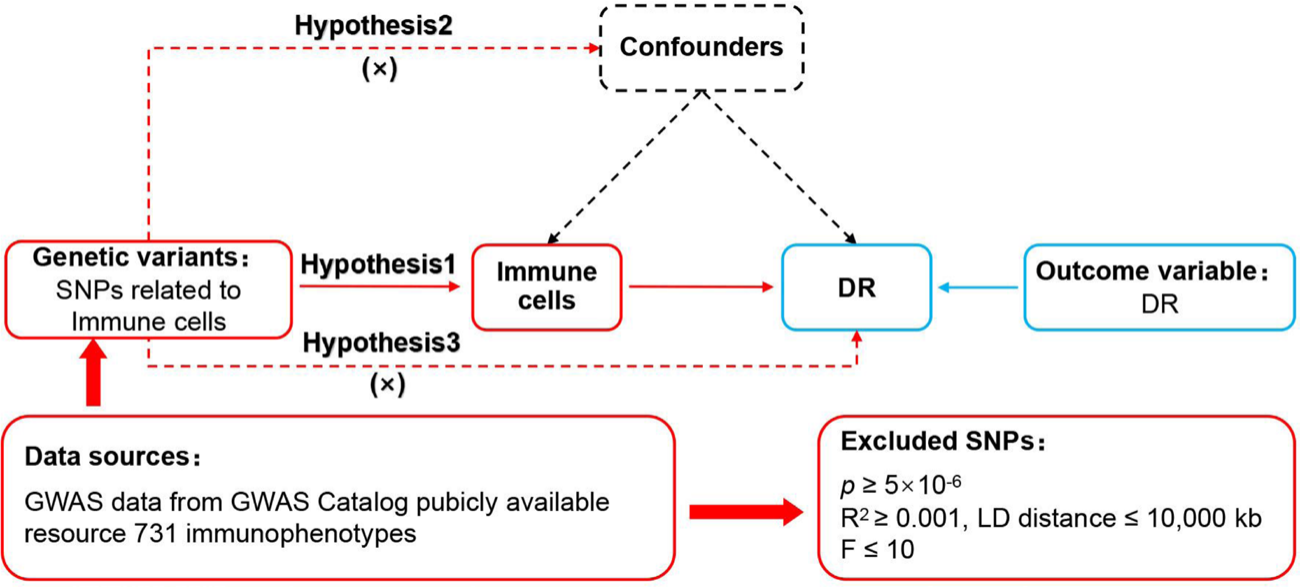
MR design for immune cells on genetic susceptibility to DR. DR = diabetic retinopathy; MR = Mendelian randomization.

The MR study of immune cells and DR are based on 3 fundamental assumptions,^[19]^ ensuring the scientific rigor and robustness of the research^[20]^ (Fig. 1). The association assumption requires that single nucleotide polymorphisms (SNPs) are strongly correlated with exposure. The independence assumption requires that SNPs are independent of confounding factors. The exclusivity assumption requires that SNPs only act on outcomes through exposure and not other pathways.

### 2.2. Data sources

The data on immune cells, numbered from GCST0001391 to GCST0002121, were obtained from European Bioinformatics Institute (www.ebi.ac.uk/).^[21]^ It originated from a study on 3757 Sardinian residents, investigating the impact of approximately 22 million variants on 731 immune cell traits, which included 118 absolute cell counts, 389 mean fluorescence intensities of surface antigens, 32 morphological parameters, and 192 relative counts.

The data on DR numbered finngen_R9_DM_ RETINOPATHY_EXMORE were acquired from FinnGen (www.finngen.fi/fi).^[22]^ The definition of DR in FinnGen is as follows: a chronic, pathological complication associated with diabetes mellitus, where retinal damages are incurred due to microaneurysms in the vasculature of the retina, progressively leading to abnormal blood vessel growth, and swelling and leaking of fluid from blood vessels, resulting in vision loss or blindness. FinnGen provided GWAS data of DR for 319,046 Europeans, including 10,413 in the experimental group and 308,633 in the control group. As European Bioinformatics Institute and FinnGen were publicly available, the study did not require additional ethical approval.

### 2.3. Selection of genetic instrument variables

First, we restricted *P* < 5 × 10^–6^ to search for SNPs closely associated with each phenotype in the dataset of immune cells to satisfy the association assumption.^[23]^ Second, we restricted *R*^2^ < 0.001 and kb = 10,000 to search for independent SNPs to exclude interference from linkage disequilibrium.^[24]^ Third, *F* > 10 was restricted to search for strongly correlated SNPs to exclude the interference of weakly correlated variables.^[25]^ *F* was calculated as *F* = [R^2^/(1–*R*^2^)]*[(N–*K*–1)/*K*]. *K* refers to the number of paired samples, N refers to the total number of samples, and *R*^2^ refers to the cumulative explained variance. Fourth, SNPs containing confounding factors were excluded by PhenoScanner and Google Scholar to fulfill the independence assumption.^[26]^ Fifth, mismatched SNPs were excluded based on the effect allele frequency when adjusting the allele orientation of the exposure and outcome.^[27]^ Sixth, significantly biased SNPs (*P* < .05) were excluded using the MR-Pleiotropy Residual Sum and Outlier method to ensure the correctness of causal inference.

### 2.4. Data analysis

The STROBE-MR was used as a guiding methodology.^[28]^ R 4.3.1 with the TwoSampleMR (0.5.7) program package installed was used to perform all operations for MR analysis. Inverse variance weighted was set as the main assessment tool because it allows for unbiased causal analysis without pleiotropy. The weighted median, which is sensitive to outliers, and MR-Egger, which analyzes data in the presence of pleiotropy, were set as secondary assessment tools. MR-Egger was also used to analyze horizontal pleiotropy, which was required to satisfy the assumption of exclusivity (*P* ≥ .05). Cochran *Q* and leave-one-out methods were used to assess heterogeneity and sensitivity, respectively. The results showed no heterogeneity when *P* ≥ .05, and were robust when the combined effect sizes were not significantly altered.

## 3. Results

### 3.1. Two-sample MR analysis

**Figure 2.**
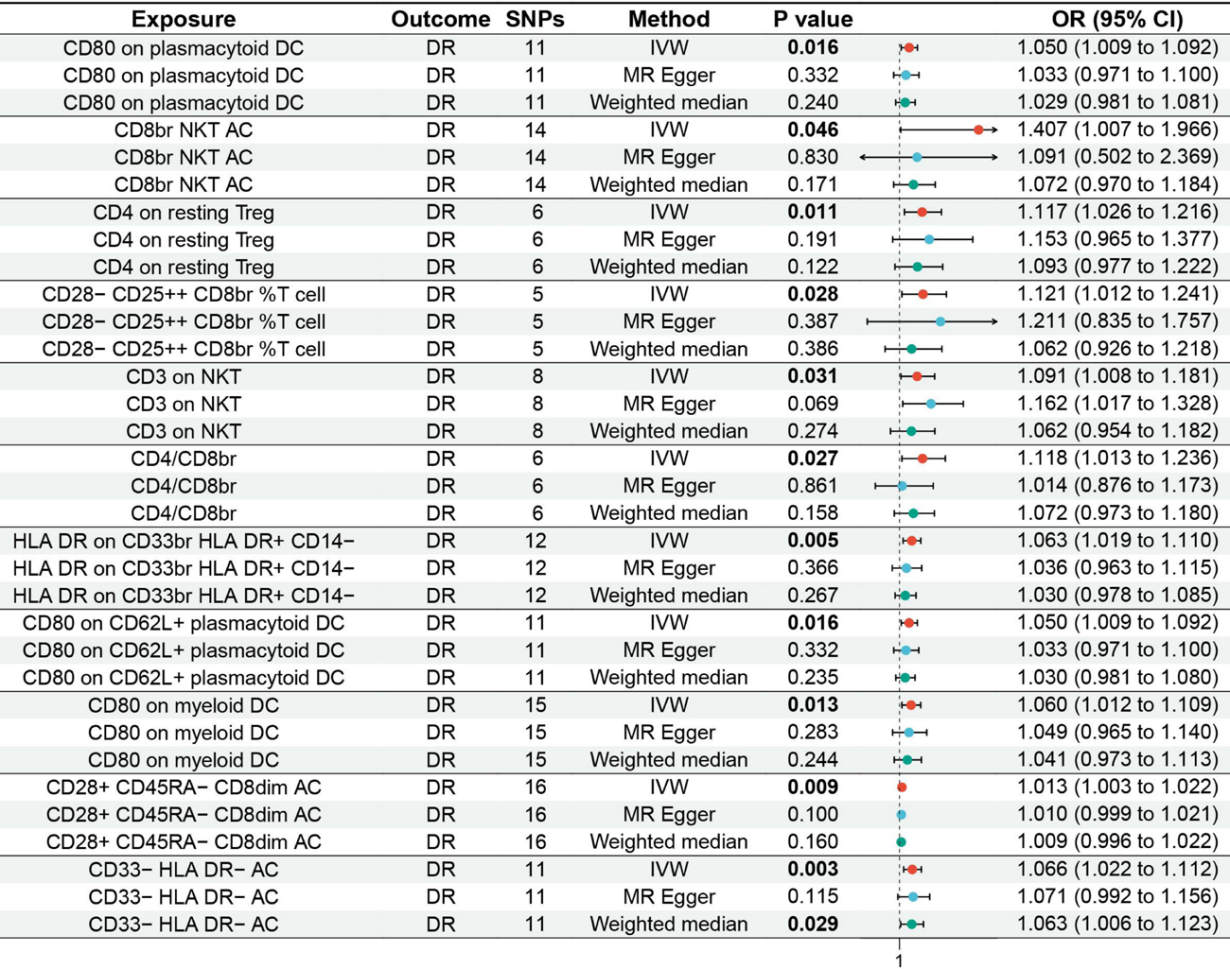
Forest plot of MR analysis for immune cells on genetic susceptibility to DR. DR = diabetic retinopathy; MR = Mendelian randomization.

**Figure 3.**
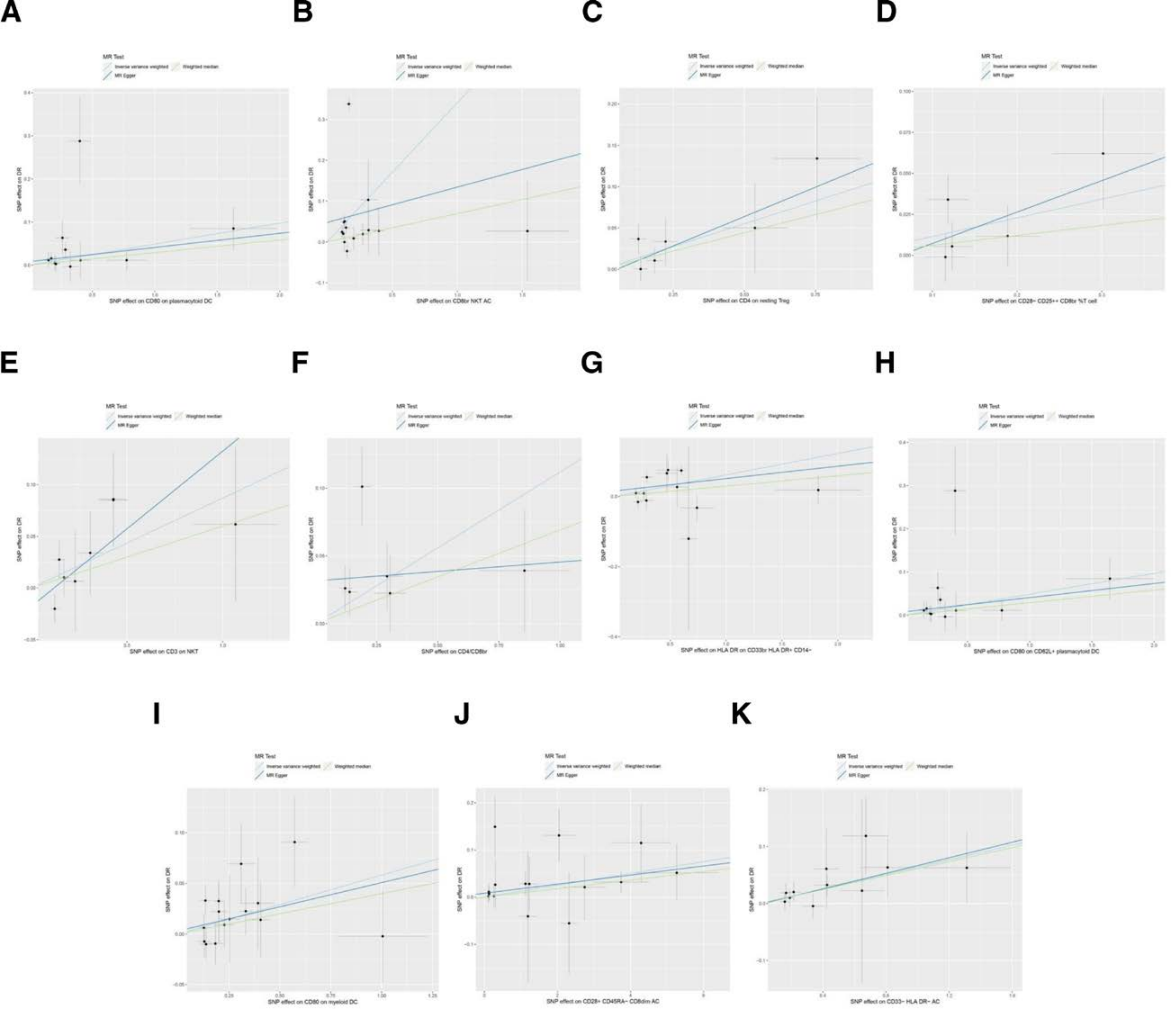
Scatter plot of MR analysis for immune cells on genetic susceptibility to DR. (A) CD80 on plasmacytoid DC on DR; (B) CD8br NKT AC on DR; (C) CD4 on resting Treg on DR; (D) CD28− CD25++ CD8br %T cell on DR; (E) CD3 on NKT on DR; (F) CD4/CD8br on DR; (G) HLA DR on CD33br HLA DR+ CD14− on DR; (H) CD80 on CD62L+ plasmacytoid DC on DR; (I) CD80 on myeloid DC on DR; (J) CD28+ CD45RA− CD8dim AC on DR; (K) CD33− HLA DR− AC on DR. DR = diabetic retinopathy; MR = Mendelian randomization.

MR analysis reported 11 immune cells associated with an increased genetic susceptibility to DR, as shown in Tables S1 to S11, Supplemental Digital Content, https://links.lww.com/MD/Q167. Among them, 4 T cell related phenotypes were significantly associated, including CD4 on resting Treg (odds ratio [OR] 1.117, 95% confidence interval [CI] 1.026–1.216, *P* = .011), CD4/CD8br (OR 1.118, 95% CI 1.013–1.236, *P* = .027), CD28+ CD45RA− CD8dim AC (OR 1.013, 95% CI 1.003–1.022, *P* = .009), and CD28− CD25++ CD8br %T cell (OR 1.121, 95% CI 1.012–1.241, *P* = .028). Two NKT cell related phenotypes were also identified, including CD8br NKT AC (OR 1.407, 95% CI 1.007–1.966, *P* = .046) and CD3 on NKT (OR 1.091, 95% CI 1.008–1.181, *P* = .031). Three dendritic cell related phenotypes showed significant associations, including CD80 on plasmacytoid DC (OR 1.050, 95% CI 1.009–1.092, *P* = .016), CD80 on CD62L+ plasmacytoid DC (OR 1.050, 95% CI 1.009–1.092, *P* = .016), and CD80 on myeloid DC (OR 1.060, 95% CI 1.012–1.109, *P* = .013). Additionally, one monocyte related phenotype ‒ HLA DR on CD33br HLA DR+ CD14− (OR 1.063, 95% CI 1.019–1.110, *P* = .005) and one myeloid cell related phenotype ‒ CD33− HLA DR− AC (OR 1.066, 95% CI 1.022–1.112, *P* = .003) were significantly associated with increased genetic susceptibility to DR. The corresponding forest and scatter plots are shown in Figures 2 and 3, respectively. No evidence of horizontal pleiotropy was detected by MR-Egger (*P* ≥ .05), as presented in Table S12, Supplemental Digital Content, https://links.lww.com/MD/Q167.

### 3.2. Heterogeneity and sensitivity analysis

**Figure 4.**
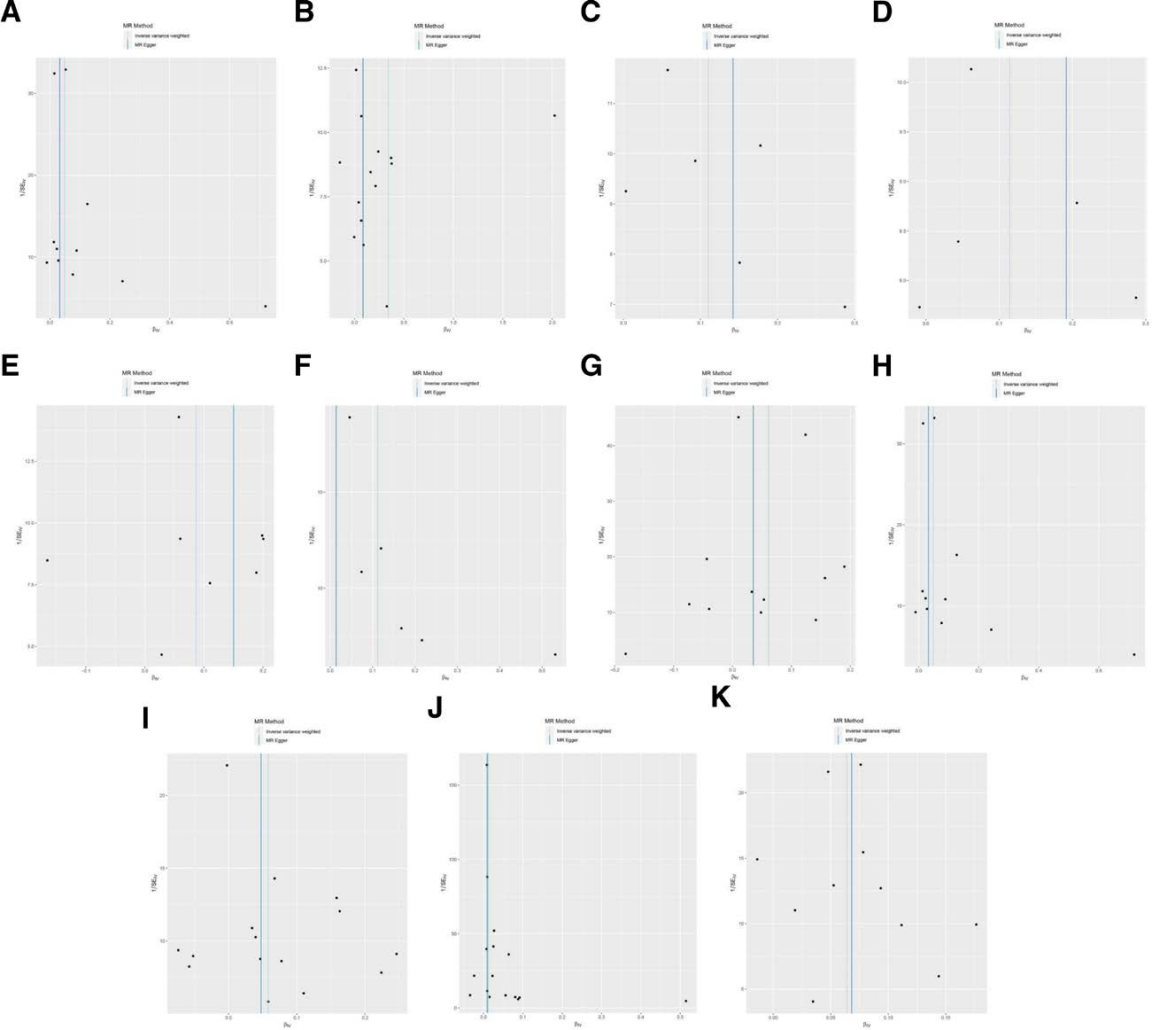
Funnel plot of MR analysis for immune cells on genetic susceptibility to DR. (A) CD80 on plasmacytoid DC on DR; (B) CD8br NKT AC on DR; (C) CD4 on resting Treg on DR; (D) CD28− CD25++ CD8br %T cell on DR; (E) CD3 on NKT on DR; (F) CD4/CD8br on DR; (G) HLA DR on CD33br HLA DR+ CD14− on DR; (H) CD80 on CD62L+ plasmacytoid DC on DR; (I) CD80 on myeloid DC on DR; (J) CD28+ CD45RA− CD8dim AC on DR; (K) CD33− HLA DR− AC on DR. DR = diabetic retinopathy; MR = Mendelian randomization.

**Figure 5.**
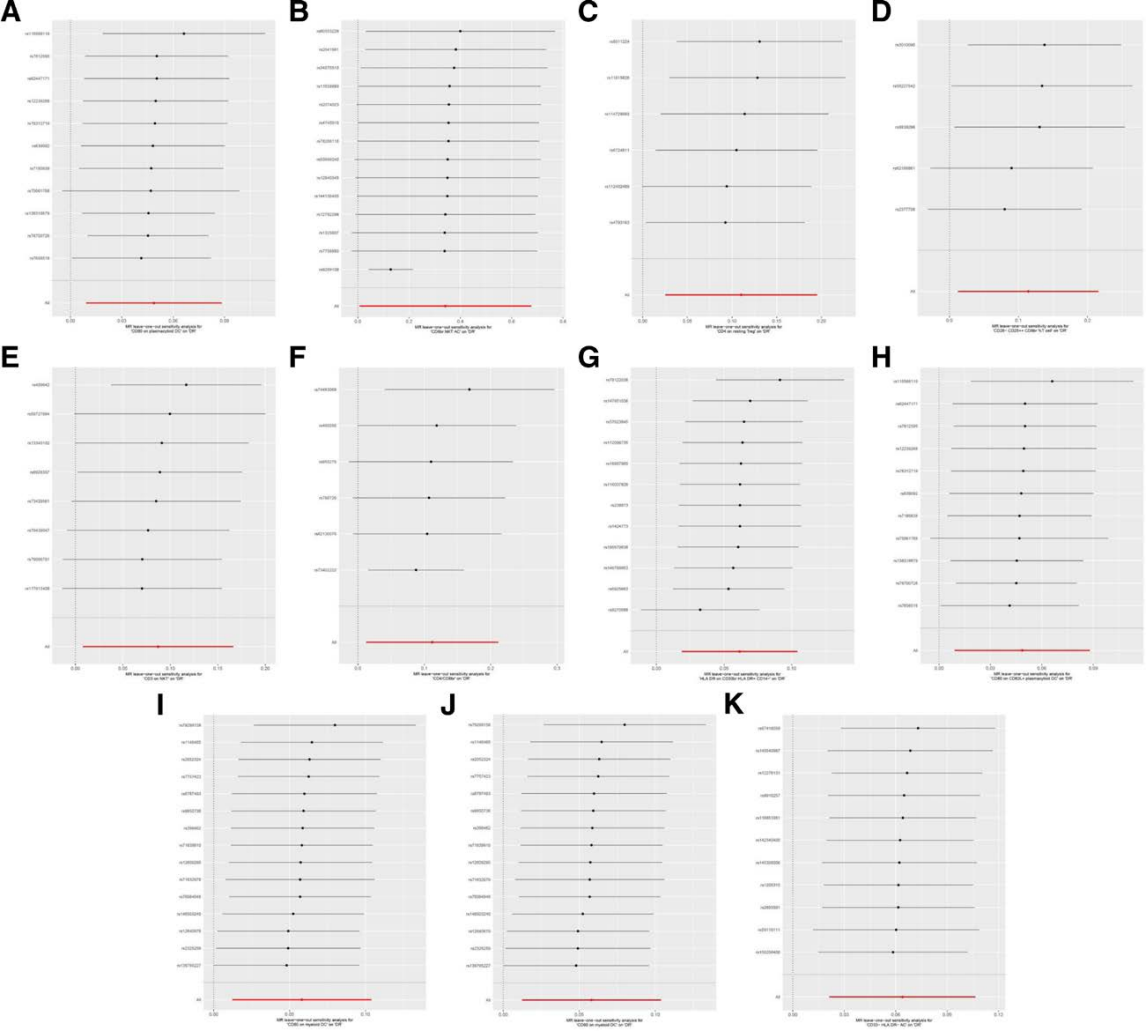
Leave-one-out sensitivity analysis for immune cells on genetic susceptibility to DR. (A) CD80 on plasmacytoid DC on DR; (B) CD8br NKT AC on DR; (C) CD4 on resting Treg on DR; (D) CD28− CD25++ CD8br %T cell on DR; (E) CD3 on NKT on DR; (F) CD4/CD8br on DR; (G) HLA DR on CD33br HLA DR+ CD14− on DR; (H) CD80 on CD62L+ plasmacytoid DC on DR; (I) CD80 on myeloid DC on DR; (J) CD28+ CD45RA− CD8dim AC on DR; (K) CD33− HLA DR− AC on DR. DR = diabetic retinopathy; MR = Mendelian randomization.

Cochran *Q* showed no heterogeneity in the results (*P *≥ .05) except for CD8br NKT AC (*P* < .001) and HLA DR on CD33br HLA DR+ CD14− (*P* = .002), as shown in Table S13, Supplemental Digital Content, https://links.lww.com/MD/Q167 and Figure 4. Sensitivity analysis suggested that these results were robust, as shown in Figure 5.

## 4. Discussion

Diabetic retinopathy is the leading cause of irreversible damage to adult vision and is one of the major public health problems threatening human health.^[29]^ Previous studies indicated a potential association between changes in immune cell subsets and the onset or progression of DR.^[30]^ However, the precise impact of different immune cells on DR remains unclear due to the limited research base. To our knowledge, this marks the first MR analysis evaluating the causal effects of 731 immune cell phenotypes on DR, employing large-scale GWAS data to comprehensively elucidate the role of diverse immune cells in DR. The results reveal that CD80 on plasmacytoid DC, CD80 on CD62L+ plasmacytoid DC, CD80 on myeloid DC, CD8br NKT AC, CD3 on NKT, CD4 on resting Treg, CD4/CD8br, HLA DR on CD33br HLA DR+ CD14−, CD28+ CD45RA− CD8dim AC, CD28− CD25++ CD8br %T cell, and CD33− HLA DR− AC are associated with an increased genetic susceptibility to DR. Although there was heterogeneity in the results for CD8br NKT AC and HLA DR on CD33br HLA DR+ CD14−, sensitivity analysis indicated the robustness of all findings.

CD80 on plasmacytoid DC refers to plasmacytoid dendritic cells with CD80 expression on its surface. CD80 on CD62L+ plasmacytoid DC refers to plasmacytoid dendritic cell with CD80 and CD62L expression. CD80 on myeloid DC refers to myeloid dendritic cells with expression of CD80 on their surface. These phenotypes point to an association between dendritic cells, CD80, and CD62L with the risk of DR. Plasmacytoid dendritic cells, as a specialized subpopulation of dendritic cells, act as antigen-presenting cells, contributing to T-lymphocyte responses, immunomodulation, plasma cell differentiation, and antibody secretion.^[31]^ Myeloid dendritic cells possess a unique antigen-presenting function, regulating the immune response by presenting antigens to T cells and providing co-stimulatory signals.^[32]^ A prior study found that in streptozotocin-induced diabetic mice, myeloid-derived dendritic cells secreted significantly more pro-inflammatory factors such as interleukin-1β and tumor necrosis factor-α, suggesting that dendritic cell may be involved in the chronic inflammatory process of diabetes and its complications.^[33]^ CD80 is primarily expressed on the surface of antigen-presenting cells, such as dendritic cells, B cells, and macrophages, and is involved in the activation and proliferation of T cells by transmitting second signals through binding to CD28.^[34]^ CD62L, also known as L-selectin, functions as a type I transmembrane glycoprotein and cell adhesion molecule crucial for mediating leukocyte adhesion and migration.^[35]^ A UK clinical study demonstrated that serum L-selectin levels were significantly higher in patients with DR compared to the nondiabetic population (992 ng/mL vs 828 ng/mL).^[36]^ Similar findings were obtained in a Turkish study, which noted higher serum sL-selectin concentrations in patients with DR compared to a healthy population at baseline (36.5 ng/mL vs 11.4 ng/mL).^[37]^ Another Saudi study further confirmed higher L-selectin levels in patients with DR compared to type 2 diabetes (T2D) patients without comorbidities (1765.42 ng/mL vs 1541.08 ng/mL).^[38]^ It suggests that L-selectin (CD62L) may play a non-negligible role in diabetes complicating DR. These findings indirectly support the notion that CD80 on plasmacytoid DC, CD80 on CD62L+ plasmacytoid DC, and CD80 on myeloid DC are potential risk factors for DR.

CD8br NKT AC refers to the absolute count of natural killer T cells with high expression of CD8. CD3 on NKT refers to natural killer T cell with CD3 expression on their surface. NKT is a specialized class of lymphocytes with innate and adaptive features of the immune system.^[38]^ It regulates both inflammatory responses and immune responses by secreting cytokines as well as directly kills tumor cells and infectious pathogens.^[39]^ Although there is no direct evidence confirming the relationship between NKT and DR, prior studies have found that patients with new-onset T2D have a significantly higher frequency of NKG2D+ NK cells and NKT cells compared to the healthy population.^[40]^ It suggests a potential role of NKT cells in diabetes and its complications. CD3, as a transmembrane signaling protein, transmits external antigenic signals and regulates T-cell function by binding to T-cell receptors.^[41]^ A clinical study in Slovenia found that the number of CD3+ cells in the fibrovascular membrane of active proliferative DR was significantly higher than that of quiescent proliferative DR, suggesting that CD3 is associated with the active phase of DR.^[42]^ CD8 is a T-cell surface marker that is crucial for immune cells to recognize and kill infected cells.^[43]^ An animal study found that STZ-induced diabetic mice had a significantly increased frequency of interferon-γ+ CD8 T cells in bone marrow (49.0 ± 2.61 vs 34.23 ± 1.19) and splenic tissues (31.75 ± 3.05 vs 19.65 ± 0.68) compared to normal mice.^[44]^ A Chinese clinical study also revealed a significantly higher percentage of CD8+ cells in the conjunctiva of patients with T2D compared to the healthy population.^[45]^ It suggests that CD8 may increase the risk of complications by promoting diabetes progression. These pieces of evidence support the association of CD3, CD8, and NKT with diabetes or DR, pointing to CD8br NKT AC and CD3 on NKT as potential risk factors for DR.

CD4 on resting Treg refers to a resting Treg with CD4 expression on surface. CD4/CD8br refers to a subset of T cells with CD4 expression and high CD8 expression on surface. Naive CD4+ T cells differentiate into Th1, Th2, and Treg subtypes upon binding to antigen-major histocompatibility complexes, thereby regulating innate and adaptive immunity.^[46]^ A Chinese clinical study demonstrated a significantly higher percentage of peripheral blood CD4+ CXCR5+ PD-1+ Tfh cells in patients with non-proliferative DR and proliferative DR compared to healthy groups.^[47]^ Another Slovenian study showed that the number of CD4+ cells in the fibrovascular membrane was significantly increased in active proliferative DR compared to quiescent proliferative DR.^[42]^ They support the association of CD4+ T cells with DR and its active phase. Treg, as one of the major subtypes of CD4+ T cells, also plays a role in diabetes and its complications. A clinical study of type 1 diabetes (T1D) showed that children with new-onset T1D had a higher frequency of conventional Treg cells than autoantibodies-negative children (7.0% vs 4.7%).^[48]^ Another study on T2D showed that T2D patients exhibited significantly higher levels of interleukin-17 expression in Foxp3-CD4T cells and Foxp3+ Treg cells compared to non-T2D patients.^[49]^ These findings support the involvement of Treg cells in the pathogenesis of T1D and T2D. Considering that diabetes is the most significant risk factor for DR, it can be inferred that Treg cells may play a role in the pathogenesis of DR. These pieces evidence support CD4 on resting Treg as a potential risk factor for DR. Additional evidence suggests that the percentage of both CD4+ T cells and CD8+ T cells in the conjunctiva of patients with T2D is significantly higher than in the healthy population,^[45]^ pointing to CD4/CD8br as a risk factor for DR.

HLA DR on CD33br HLA DR+ CD14− refers to cells with high CD33 expression, HLA DR expression, and CD14 non-expression. HLA DR and CD33 are involved in inflammatory responses and immune responses.^[50,51]^ The HLA-DR molecule is an major histocompatibility complex class-II molecule primarily found on the surface of antigen-presenting cells. A case–control study in Mexico showed that HLA-DR7 was significantly elevated in patients with non-proliferative DR compared to the healthy population (OR 3.49, 95% CI 1.29–9.4).^[52]^ A cross-sectional study conducted in the United States found that patients with proliferative DR had significantly higher frequencies of HLA-DQ4 (75.0% vs 40.0%) and HLA-DR4/HLA-DQ4 haplotypes (65.0% *vs* 28.0%) compared to patients without DR.^[53]^ These pieces of evidence suggest a role for HLA-DR in the pathogenesis of DR. CD33 is mainly expressed on myeloid cells and implicated in the pathogenesis of T2D. A Mexican study showed that T2D patients had a higher frequency of CD33+ HLA-DR−/low mononuclear MDSCs than the general population at the same baseline.^[54]^ Another Chinese study also noted an increased frequency of CD11b+/CD33+ MDSCs in the peripheral blood of T2D patients compared to the healthy individuals.^[55]^ These findings support that HLA DR and CD33 are directly or indirectly associated with DR, pointing to HLA DR on CD33br HLA DR+ CD14− as a potential risk factor for DR.

CD28+ CD45RA− CD8dim AC refers to the absolute count of cells with CD28 expression, CD45RA non-expression, and CD8 low-expression. CD28, primarily expressed on activated T-cells, regulates T-cell proliferation and differentiation.^[56]^ A Tunisian study found that rs1879877 and rs3181096 in the CD28 gene were associated with an increased risk of T1D,^[57]^ suggesting that CD28 may be involved in the pathogenesis of T1D. Established evidence supports the association of CD28 and CD8 with diabetes risk, implying that CD28+ CD45RA− CD8dim AC is a potential risk factor for DR. Additionally, the study found that CD28− CD25++ CD8br %T cells were associated with an increased risk of DR CD28− CD25++ CD8br %T cell refers to the proportion of cells within the total T cell population that have CD28 non-expression as well as CD25 and CD8 high expression. CD25, also known as the interleukin-2 receptor alpha chain, plays an important role in regulating Treg and effector T cells, as well as in cell apoptosis.^[58]^ Although there is no direct evidence associating CD25 with DR, some reports support the association of CD25 with T1D. A UK clinical study showed that sCD25 levels were significantly increased in T1D patients compared to the general population.^[59]^ Another French study found that patients with new-onset T1D had a significantly higher frequency of CD25+ MAIT cells than the normal population.^[60]^ Current evidence points to the association of CD25 and CD8 with diabetes risk, suggesting that CD28− CD25++ CD8br %T cell may be a risk factor for DR.

Our findings are consistent with earlier evidence supporting the association between dendritic cells, T cells, and NKT cells and the risk of DR, highlighting the role of chronic low-grade inflammation and immune dysregulation in diabetic retinal vascular injury. Distinctly, this study identifies specific immune cell phenotypes that increase the genetic susceptibility to DR, including 4 T cell-related phenotypes, 2 NKT cell-related phenotypes, 3 dendritic cell-related phenotypes, one monocyte-related phenotype, and one myeloid cell-related phenotype. The identification of immune phenotypes such as CD80-expressing dendritic cells, CD4+ T cells, and CD8+ T cells underscores their potential involvement in DR and provides valuable insights for the development of biomarkers and therapeutic targets. To enhance the translational relevance of these findings, future studies should prioritize validation in well-characterized diabetic cohorts, including both treated and untreated populations. Longitudinal cohort studies or nested case–control designs within randomized controlled trials could assess whether individuals with genetically or phenotypically elevated levels of these immune cell subsets exhibit higher DR incidence or faster progression. Moreover, immune profiling of peripheral blood or ocular tissues using flow cytometry or single-cell sequencing could be applied to evaluate the consistency between genetically predicted immune cell patterns and their actual expression in diabetic patients. These strategies offer a pathway to validate genetic findings at the clinical level by linking genetically predicted immune cell patterns with their actual expression in diabetic patients. This could not only clarify the immunological mechanisms underlying DR but also facilitate the development of immune-based biomarkers and therapeutic strategies, ultimately supporting more precise, patient-centered, and cost-effective DR care.

While this MR analysis enriches the genetic evidence for a causal relationship between immune cell phenotypes and DR, it has inevitable limitations. First, the study mainly reveals the relationship between different immune cells and genetic susceptibility to DR in individuals of European ancestry, and therefore cannot be extrapolated to African or Asian populations. Second, MR reflects genetic predisposition to immune cell phenotypes rather than actual immune cell counts or activity levels in vivo, limiting its ability to capture dynamic immune responses in DR. Third, the lack of subtype-specific data in DR-related GWAS impedes the analysis of differential effects of immune cell phenotypes on active proliferative versus quiescent proliferative DR. Fourth, although 11 immune cell phenotypes were identified as genetically associated with DR, the underlying biological mechanisms remain unclear. Fifth, heterogeneity observed in the MR results for CD8br NKT AC and HLA DR on CD33br HLA DR+ CD14− may introduce potential bias. Finally, although no horizontal pleiotropy was detected using MR-Egger regression, the possibility of undetected or residual pleiotropy cannot be completely excluded, which may affect the causal estimates. We expect future studies to address these limitations in several ways. First, by continuously enriching genomic databases across diverse populations to promote equitable health research and applicability. Second, by conducting multicenter, large-sample clinical studies to investigate the specific roles of different immune cell phenotypes in DR and its subtypes. Third, by validating the causal effects and mechanisms of the 11 identified immune cell phenotypes in animal models, thereby strengthening the biological evidence underlying our findings.

## 5. Conclusion

The MR analysis revealed that CD80 on plasmacytoid DC, CD80 on CD62L+ plasmacytoid DC, CD80 on myeloid DC, CD8br NKT AC, CD3 on NKT, CD4 on resting Treg, CD4/CD8br, HLA DR on CD33br HLA DR+ CD14−, CD28+ CD45RA− CD8dim AC, CD28− CD25++ CD8br %T cell, and CD33− HLA DR− AC were associated with an increased genetic susceptibility to DR. These findings provide a new perspective on the pathogenesis and drug development of DR. However, due to limited clinical evidence, further biological studies are required to elucidate the specific mechanisms.

## Author contributions

**Conceptualization:** Pengfei Jiang, Pei Liu.

**Data curation:** Gang Hu, Xinyu Yang.

**Formal analysis:** Yunfeng Yu.

**Methodology:** Donghua Liu, Yunfeng Yu.

**Supervision:** Pengfei Jiang, Pei Liu.

**Writing – original draft:** Pengfei Jiang, Yunfeng Yu, Jingyi Wu, Gang Hu, Xinyu Yang, Pei Liu.

**Writing – review & editing:** Donghua Liu, Jingyi Wu, Pei Liu.

## Supplementary Material


