# Supplementary material for: Immune cells in diabetic retinopathy: A Mendelian randomization study
Source: Medicine (Baltimore). 2025 Oct 10;104(41):e44549. doi: 10.1097/MD.0000000000044549 (PMC12517842; doi:10.1097/MD.0000000000044549)
Supplement: Supplementary file 1 [file medi-104-e44549-s001.pdf]

Supplementary Table S1 Information of identified SNPs in exposure (CD80 on plasmacytoid DC) and outcomes (DR).

				Exposure (CD80 on plasmacytoid DC)			Outcome (DR)				
	**SNP**	**EA**	**OA**	**β**	**SE**	***p* value**	**Case**	**Control**	**β**	**SE**	***p* v**[illegible]
1	rs115568119	T	C	-0.7747	0.1594	1.24E-06	10,413	308,633	-0.0113864	0.0238916	0.63[illegible]
2	rs12239268	A	G	-0.2001	0.04166	1.64E-06	10,413	308,633	-0.00460907	0.0182044	0.80[illegible]
3	rs138318679	T	G	0.2612	0.05496	2.11E-06	10,413	308,633	0.0632826	0.0370765	0.08[illegible]
4	rs62447171	A	G	-0.2093	0.03307	2.84E-10	10,413	308,633	-0.00270495	0.017694	0.87[illegible]
5	rs639092	G	A	0.1502	0.03257	4.16E-06	10,413	308,633	0.0114634	0.0190952	0.54[illegible]
6	rs7185638	A	G	0.172	0.03451	6.58E-07	10,413	308,633	0.0153094	0.0159391	0.33[illegible]
7	rs75061768	A	G	1.63	0.3494	3.24E-06	10,413	308,633	0.0848413	0.0495425	0.08[illegible]
8	rs7658518	A	G	-0.2855	0.03051	1.58E-20	10,413	308,633	-0.0357403	0.0173244	0.03[illegible]
9	rs7812595	T	G	0.324	0.06425	4.86E-07	10,413	308,633	-0.00337882	0.0347745	0.92[illegible]
10	rs78312719	A	G	-0.406	0.08858	4.76E-06	10,413	308,633	-0.0112928	0.0424364	0.79[illegible]
11	rs78700726	C	G	-0.4002	0.08703	4.43E-06	10,413	308,633	-0.288019	0.100701	0.004[illegible]

SNP, single nucleotide polymorphism; EA, effect allele; OA, other allele; SE, standard error; DR, diabetic retinopathy.

Supplementary Table S2 Information of identified SNPs in exposure (CD8br NKT AC) and outcomes (DR).

				Exposure (CD8br NKT AC)			Outcome (DR)				
	SNP	EA	OA	β	SE	*p* value	Case	Control	β	SE	*p* v[illegible]
1	rs11658693	G	A	-0.2038	0.03962	2.84E-07	10,413	308,633	-0.00872786	0.0279878	0.75[illegible]
2	rs12792298	T	C	-0.3155	0.06667	2.30E-06	10,413	308,633	-0.103201	0.0974411	0.28[illegible]
3	rs12940345	T	C	0.1138	0.02473	4.33E-06	10,413	308,633	0.0242936	0.0143758	0.09[illegible]
4	rs1325907	A	G	-0.1298	0.02573	4.73E-07	10,413	308,633	-0.0481191	0.0144101	0.000[illegible]
5	rs144156455	C	T	0.3176	0.06734	2.49E-06	10,413	308,633	0.0288364	0.0564529	0.60[illegible]
6	rs2041561	C	G	0.1523	0.03234	2.59E-06	10,413	308,633	-0.0225222	0.0172451	0.1[illegible]
7	rs2074023	T	C	-0.1237	0.02486	6.76E-07	10,413	308,633	-0.0201678	0.0146254	0.16[illegible]
8	rs34575510	T	C	0.2742	0.05762	2.03E-06	10,413	308,633	0.0190765	0.0257895	0.4[illegible]
9	rs4745010	G	A	0.1325	0.02737	1.35E-06	10,413	308,633	-0.00027893	0.0223358	0.99[illegible]
10	rs55699240	C	G	0.1451	0.03161	4.57E-06	10,413	308,633	0.0349973	0.0156756	0.02[illegible]
11	rs60553229	A	G	-1.538	0.32	1.60E-06	10,413	308,633	-0.0266071	0.123735	0.82[illegible]
12	rs7756993	C	T	-0.1338	0.02858	2.96E-06	10,413	308,633	-0.0503939	0.0152249	0.000[illegible]
13	rs78268116	T	C	-0.3964	0.0389	4.65E-24	10,413	308,633	-0.0271149	0.0603282	0.65[illegible]
14	rs9269109	C	T	0.1671	0.03018	3.31E-08	10,413	308,633	0.338345	0.0156839	3.24[illegible]

SNP, single nucleotide polymorphism; EA, effect allele; OA, other allele; SE, standard error; DR, diabetic retinopathy.

Supplementary Table S3 Information of identified SNPs in exposure (CD4 on resting Treg) and outcomes (DR).

				Exposure (CD4 on resting Treg)			Outcome (DR)				
	**SNP**	**EA**	**OA**	**β**	**SE**	***p* value**	**Case**	**Control**	**β**	**SE**	***p* v[illegible]**
1	rs112402469	T	C	0.7551	0.1561	1.39E-06	10,413	308,633	0.134126	0.0743152	0.07[illegible]
2	rs114729093	C	T	-0.5372	0.114	2.57E-06	10,413	308,633	-0.0501087	0.0545022	0.35[illegible]
3	rs11615628	A	G	-0.1847	0.03074	2.11E-09	10,413	308,633	-0.0106153	0.0158328	0.50[illegible]
4	rs4793163	A	G	-0.128	0.02686	1.98E-06	10,413	308,633	-0.0367819	0.0184155	0.04[illegible]
5	rs6724611	A	T	-0.2234	0.04413	4.42E-07	10,413	308,633	-0.0336876	0.0285282	0.23[illegible]
6	rs8011224	A	T	0.1358	0.02708	5.65E-07	10,413	308,633	0.00043668	0.0146766	0.97[illegible]

SNP, single nucleotide polymorphism; EA, effect allele; OA, other allele; SE, standard error; DR, diabetic retinopathy.

Supplementary Table S4 Information of identified SNPs in exposure (CD28- CD25++ CD8br %T cell) and outcomes (DR).

				Exposure (CD28- CD25++ CD8br %T cell)			Outcome (DR)				
	**SNP**	**EA**	**OA**	**β**	**SE**	***p* value**	**Case**	**Control**	**β**	**SE**	***p* v[illegible]**
1	rs2377708	C	A	0.1185	0.02489	2.02E-06	10,413	308,633	0.0339933	0.0151422	0.02[illegible]
2	rs3010090	T	C	0.1155	0.02416	1.82E-06	10,413	308,633	-0.00101525	0.0149407	0.94[illegible]
3	rs58227542	A	T	0.1889	0.03767	5.62E-07	10,413	308,633	0.0116628	0.018642	0.53[illegible]
4	rs62166861	A	G	0.3014	0.05959	4.46E-07	10,413	308,633	0.062131	0.0343194	0.07[illegible]
5	rs9839296	G	A	0.1233	0.0248	7.03E-07	10,413	308,633	0.00544387	0.0146918	0.71[illegible]

SNP, single nucleotide polymorphism; EA, effect allele; OA, other allele; SE, standard error; DR, diabetic retinopathy.

Supplementary Table S5 Information of identified SNPs in exposure (CD3 on NKT) %T cell) and outcomes (DR).

				Exposure (CD3 on NKT) %T cell)			Outcome (DR)				
	**SNP**	**EA**	**OA**	**β**	**SE**	***p* value**	**Case**	**Control**	**β**	**SE**	***p* v[illegible]**
1	rs117913408	G	A	0.4285	0.07737	3.31E-08	10,413	308,633	0.0851141	0.0451534	0.05[illegible]
2	rs13345102	T	C	0.1706	0.03205	1.10E-07	10,413	308,633	0.0102209	0.0182254	0.5[illegible]
3	rs489642	T	G	-0.122	0.02649	4.31E-06	10,413	308,633	0.0201567	0.0143814	0.1[illegible]
4	rs59727684	T	C	-1.067	0.2238	1.92E-06	10,413	308,633	-0.0616054	0.074673	0.4[illegible]
5	rs6928357	A	C	0.2289	0.0486	2.58E-06	10,413	308,633	0.0064913	0.0490212	0.8[illegible]
6	rs73429561	C	A	0.308	0.0671	4.60E-06	10,413	308,633	0.0339498	0.0407216	0.4[illegible]
7	rs79086781	T	C	0.4289	0.06851	4.36E-10	10,413	308,633	0.0860303	0.045858	0.06[illegible]
8	rs79439047	A	G	-0.1456	0.03122	3.24E-06	10,413	308,633	-0.0274872	0.018223	0.1[illegible]

SNP, single nucleotide polymorphism; EA, effect allele; OA, other allele; SE, standard error; DR, diabetic retinopathy.

Supplementary Table S6 Information of identified SNPs in exposure (CD4/CD8br) and outcomes (DR).

				Exposure (CD4/CD8br)			Outcome (DR)				
	**SNP**	**EA**	**OA**	**β**	**SE**	***p* value**	**Case**	**Control**	**β**	**SE**	***p* v[illegible]**
1	rs495055	G	A	0.3049	0.06527	3.10E-06	10,413	308,633	0.0225194	0.0281093	0.4[illegible]
2	rs62130076	A	G	-0.121	0.02596	3.26E-06	10,413	308,633	-0.0261613	0.0166092	0.1[illegible]
3	rs73402222	C	T	0.1905	0.03406	2.39E-08	10,413	308,633	0.10116	0.0291105	0.000[illegible]
4	rs74493069	A	G	0.8545	0.1858	4.36E-06	10,413	308,633	0.0391602	0.0452101	0.3[illegible]
5	rs798726	C	T	-0.1395	0.03005	3.56E-06	10,413	308,633	-0.0234425	0.0176421	0.1[illegible]
6	rs955275	G	A	0.2925	0.06149	2.04E-06	10,413	308,633	0.0350364	0.0242364	0.1[illegible]

SNP, single nucleotide polymorphism; EA, effect allele; OA, other allele; SE, standard error; DR, diabetic retinopathy.

Supplementary Table S7 Information of identified SNPs in exposure (HLA DR on CD33br HLA DR+ CD14-) and outcomes (DR).

				Exposure (HLA DR on CD33br HLA DR+ CD14-)			Outcome (DR)				
	**SNP**	**EA**	**OA**	**β**	**SE**	***p* value**	**Case**	**Control**	**β**	**SE**	***p* v[illegible]**
1	rs112096735	T	G	-0.286	0.06037	2.36E-06	10,413	308,633	0.011395	0.0269785	0.67[illegible]
2	rs116007826	G	A	0.6643	0.07716	1.75E-17	10,413	308,633	-0.120546	0.262288	0.64[illegible]
3	rs1424773	A	T	-0.1924	0.04154	3.95E-06	10,413	308,633	-0.0102413	0.0156614	0.51[illegible]
4	rs146788953	A	G	-0.481	0.1005	1.86E-06	10,413	308,633	-0.0754829	0.0297697	0.01[illegible]
5	rs147951036	A	G	-0.7396	0.1516	1.17E-06	10,413	308,633	0.0322736	0.0377381	0.39[illegible]
6	rs16987905	G	A	-0.2637	0.05636	3.15E-06	10,413	308,633	-0.00863271	0.019243	0.6[illegible]
7	rs186879638	T	C	0.4693	0.09858	2.10E-06	10,413	308,633	0.0664069	0.0544564	0.22[illegible]
8	rs238873	G	A	0.5614	0.1033	6.26E-08	10,413	308,633	0.0270844	0.0562953	0.63[illegible]
9	rs57623845	C	A	-0.2111	0.04284	9.25E-07	10,413	308,633	0.0155245	0.0184188	0.39[illegible]
10	rs6925683	G	T	0.2904	0.05585	2.25E-07	10,413	308,633	0.0551379	0.0159364	0.000[illegible]
11	rs78122038	G	C	-1.826	0.3866	2.53E-06	10,413	308,633	-0.0184545	0.0404619	0.64[illegible]
12	rs9270588	T	C	-0.5992	0.03915	2.14E-49	10,413	308,633	-0.0742536	0.0142772	1.98[illegible]

SNP, single nucleotide polymorphism; EA, effect allele; OA, other allele; SE, standard error; DR, diabetic retinopathy.

Supplementary Table S8 Information of identified SNPs in exposure (CD80 on CD62L+ plasmacytoid DC) and outcomes (DR).

	SNP	EA	OA	Exposure (CD80 on CD62L+ plasmacytoid DC)			Outcome (DR)				
				**β**	**SE**	***p* value**	**Case**	**Control**	**β**	**SE**	***p* v[illegible]**
1	rs115568119	T	C	-0.7767	0.1594	1.17E-06	10,413	308,633	-0.0113864	0.0238916	0.63[illegible]
2	rs12239268	A	G	-0.1987	0.04166	1.95E-06	10,413	308,633	-0.00460907	0.0182044	0.80[illegible]
3	rs138318679	T	G	0.2618	0.05496	2.00E-06	10,413	308,633	0.0632826	0.0370765	0.08[illegible]
4	rs62447171	A	G	-0.2089	0.03307	3.09E-10	10,413	308,633	-0.00270495	0.017694	0.87[illegible]
5	rs639092	G	A	0.1504	0.03257	4.03E-06	10,413	308,633	0.0114634	0.0190952	0.54[illegible]
6	rs7185638	A	G	0.1724	0.03451	6.19E-07	10,413	308,633	0.0153094	0.0159391	0.33[illegible]
7	rs75061768	A	G	1.644	0.3494	2.67E-06	10,413	308,633	0.0848413	0.0495425	0.08[illegible]
8	rs7658518	A	G	-0.2821	0.03052	4.45E-20	10,413	308,633	-0.0357403	0.0173244	0.03[illegible]
9	rs7812595	T	G	0.3208	0.06426	6.29E-07	10,413	308,633	-0.00337882	0.0347745	0.92[illegible]
10	rs78312719	A	G	-0.4082	0.08858	4.24E-06	10,413	308,633	-0.0112928	0.0424364	0.79[illegible]
11	rs78700726	C	G	-0.4012	0.08702	4.19E-06	10,413	308,633	-0.288019	0.100701	0.004[illegible]

SNP, single nucleotide polymorphism; EA, effect allele; OA, other allele; SE, standard error; DR, diabetic retinopathy.

Supplementary Table S9 Information of identified SNPs in exposure (CD80 on myeloid DC) and outcomes (DR).

				Exposure (CD80 on myeloid DC)			Outcome (DR)				
	**SNP**	**EA**	**OA**	**β**	**SE**	***p* value**	**Case**	**Control**	**β**	**SE**	***p* v[illegible]**
1	rs1146465	G	A	-0.1373	0.02846	1.47E-06	10,413	308,633	0.0100928	0.0146573	0.49[illegible]
2	rs12609290	A	G	0.3923	0.08387	3.04E-06	10,413	308,633	0.0304018	0.0455633	0.50[illegible]
3	rs12640679	C	T	0.1342	0.02856	2.74E-06	10,413	308,633	0.0330071	0.014741	0.02[illegible]
4	rs139795227	C	A	-0.5725	0.0642	8.24E-19	10,413	308,633	-0.0906484	0.0441907	0.04[illegible]
5	rs146503240	T	C	0.3095	0.06674	3.70E-06	10,413	308,633	0.069276	0.0395587	0.07[illegible]
6	rs2052324	A	C	-0.1828	0.03667	6.56E-07	10,413	308,633	0.00945345	0.0204	0.64[illegible]
7	rs2325259	C	T	0.1995	0.02793	1.17E-12	10,413	308,633	0.0324805	0.0165661	0.04[illegible]
8	rs398462	C	T	-0.1266	0.02753	4.43E-06	10,413	308,633	-0.00589638	0.0144469	0.68[illegible]
9	rs6787493	C	T	0.4055	0.04516	4.79E-19	10,413	308,633	0.0139713	0.0372593	0.70[illegible]
10	rs71632979	G	A	0.3323	0.0314	1.06E-25	10,413	308,633	0.0224284	0.0232541	0.3[illegible]
11	rs71639910	A	G	0.2523	0.05465	4.07E-06	10,413	308,633	0.0146888	0.0432644	0.73[illegible]
12	rs76064946	G	T	-0.2002	0.04253	2.62E-06	10,413	308,633	-0.0220273	0.0312737	0.48[illegible]
13	rs7757423	A	G	-0.1289	0.02809	4.66E-06	10,413	308,633	0.00741821	0.0156327	0.63[illegible]
14	rs79299158	A	G	1.005	0.2197	4.91E-06	10,413	308,633	-0.00218753	0.0455445	0.96[illegible]
15	rs9650736	A	C	0.2271	0.04703	1.44E-06	10,413	308,633	0.00902946	0.0221237	0.68[illegible]

SNP, single nucleotide polymorphism; EA, effect allele; OA, other allele; SE, standard error; DR, diabetic retinopathy.

Supplementary Table S10 Information of identified SNPs in exposure (CD28+ CD45RA- CD8dim AC) and outcomes (DR).

				Exposure (CD28+ CD45RA- CD8dim AC)			Outcome (DR)				
	**SNP**	**EA**	**OA**	**β**	**SE**	***p* value**	**Case**	**Control**	**β**	**SE**	***p* v[illegible]**
1	rs11579717	A	G	-1.125	0.169	3.18E-11	10,413	308,633	-0.0284461	0.0271909	0.29[illegible]
2	rs117586381	A	G	-2.041	0.4452	4.71E-06	10,413	308,633	-0.130908	0.056598	0.02[illegible]
3	rs138232290	A	G	-2.743	0.5787	2.23E-06	10,413	308,633	-0.0210754	0.0691208	0.76[illegible]
4	rs138678985	A	G	0.2897	0.06184	2.92E-06	10,413	308,633	0.149146	0.0627392	0.01[illegible]
5	rs138915779	A	C	-1.228	0.2078	3.74E-09	10,413	308,633	-0.0281053	0.0569267	0.62[illegible]
6	rs147612560	A	G	4.289	0.8146	1.49E-07	10,413	308,633	0.114864	0.0824888	0.16[illegible]
7	rs181777922	T	C	-1.191	0.2421	9.12E-07	10,413	308,633	0.0406011	0.138574	0.76[illegible]
8	rs2585739	A	G	0.2989	0.06454	3.78E-06	10,413	308,633	0.0263109	0.0513373	0.60[illegible]
9	rs2670003	C	T	-0.1279	0.0272	2.66E-06	10,413	308,633	-0.00714998	0.0150935	0.63[illegible]
10	rs28527279	A	G	-0.1214	0.02643	4.54E-06	10,413	308,633	-0.011128	0.0173658	0.52[illegible]
11	rs4129441	C	T	-0.1161	0.02528	4.53E-06	10,413	308,633	-0.00936069	0.0158944	0.55[illegible]
12	rs541367304	A	G	-2.319	0.231	2.18E-23	10,413	308,633	0.0552674	0.107	0.60[illegible]
13	rs565801	G	T	-0.1068	0.02285	3.08E-06	10,413	308,633	-0.00161137	0.0143933	0.91[illegible]
14	rs687264	C	T	-0.2594	0.05546	3.03E-06	10,413	308,633	-0.00248776	0.0226897	0.91[illegible]
15	rs77547518	T	C	-5.264	1.141	4.07E-06	10,413	308,633	-0.0515963	0.0596555	0.38[illegible]
16	rs78228882	T	G	-3.741	0.7645	1.04E-06	10,413	308,633	-0.0321425	0.0228583	0.15[illegible]

SNP, single nucleotide polymorphism; EA, effect allele; OA, other allele; SE, standard error; DR, diabetic retinopathy.

Supplementary Table S11 Information of identified SNPs in exposure (CD33- HLA DR- AC) and outcomes (DR).

				Exposure (CD33- HLA DR- AC)			Outcome (DR)				
	**SNP**	**EA**	**OA**	**β**	**SE**	***p* value**	**Case**	**Control**	**β**	**SE**	***p* v**[illegible]
1	rs116851051	C	T	-0.648	0.1415	4.97E-06	10,413	308,633	-0.0224008	0.160198	0.88[illegible]
2	rs1205315	G	A	-0.1664	0.03605	4.19E-06	10,413	308,633	-0.0185546	0.0168282	0.27[illegible]
3	rs12276131	A	G	-0.1585	0.03347	2.35E-06	10,413	308,633	-0.00299409	0.0143819	0.83[illegible]
4	rs142340400	G	C	0.4225	0.08113	2.13E-07	10,413	308,633	0.0607595	0.0706537	0.3[illegible]
5	rs145308006	G	A	-0.8101	0.1738	3.37E-06	10,413	308,633	-0.0633262	0.0524043	0.22[illegible]
6	rs145540967	G	C	-1.312	0.2783	2.61E-06	10,413	308,633	-0.0626421	0.0607371	0.3[illegible]
7	rs150206406	T	A	0.6723	0.1447	3.61E-06	10,413	308,633	0.118635	0.0676746	0.07[illegible]
8	rs2893591	A	G	0.2146	0.04597	3.26E-06	10,413	308,633	0.020053	0.0168948	0.23[illegible]
9	rs59118111	C	G	-0.4264	0.08487	5.57E-07	10,413	308,633	-0.0324256	0.0192534	0.09[illegible]
10	rs67418059	T	C	0.3374	0.07164	2.66E-06	10,413	308,633	-0.00466584	0.0226188	0.83[illegible]
11	rs9916257	T	G	-0.1904	0.03354	1.59E-08	10,413	308,633	-0.00999436	0.0147305	0.49[illegible]

SNP, single nucleotide polymorphism; EA, effect allele; OA, other allele; SE, standard error; DR, diabetic retinopathy.

Supplementary Table S12 The results of MR-Egger intercept analysis.

Exposure	Outcome	Egger_intercept	SE	*p* value
CD80 on plasmacytoid DC	DR	0.00810333	0.012398911	0.529742097
CD8br NKT AC	DR	0.047745688	0.066597744	0.487139772
CD4 on resting Treg	DR	-0.007281402	0.018084571	0.707809715
CD28- CD25++ CD8br %T cell	DR	-0.011948272	0.027871335	0.697064373
CD3 on NKT	DR	-0.017820277	0.015612202	0.297189217
CD4/CD8br	DR	0.031662204	0.019418372	0.17832411
HLA DR on CD33br HLA DR+ CD14-	DR	0.015887246	0.018288655	0.4053807
CD80 on CD62L+ plasmacytoid DC	DR	0.008184963	0.012338714	0.523728155
CD80 on myeloid DC	DR	0.002962349	0.010131071	0.774593574
CD28+ CD45RA- CD8dim AC	DR	0.008311144	0.007384186	0.279289032
CD33- HLA DR- AC	DR	-0.001568357	0.012060863	0.899397237

DR, diabetic retinopathy.

Supplementary Table S13 The results of Cochran's Q analysis.

Exposure	Outcome	Method	Q	Q_df	Q_*p* val
CD80 on plasmacytoid DC	DR	MR Egger	12.05343187	9	0.21031381
CD80 on plasmacytoid DC	DR	Inverse variance weighted	12.62547429	10	0.245372091
CD8br NKT AC	DR	MR Egger	367.6010902	12	2.70E-71
CD8br NKT AC	DR	Inverse variance weighted	383.3461683	13	7.33E-74
CD4 on resting Treg	DR	MR Egger	3.310666756	4	0.507243982
CD4 on resting Treg	DR	Inverse variance weighted	3.472778155	5	0.627510585
CD28- CD25++ CD8br %T cell	DR	MR Egger	3.778484163	3	0.286398722
CD28- CD25++ CD8br %T cell	DR	Inverse variance weighted	4.009952208	4	0.40466064
CD3 on NKT	DR	MR Egger	6.437311336	6	0.376023888
CD3 on NKT	DR	Inverse variance weighted	7.835139537	7	0.347355512
CD4/CD8br	DR	MR Egger	6.028929822	4	0.196998169
CD4/CD8br	DR	Inverse variance weighted	10.03609155	5	0.074219043
HLA DR on CD33br HLA DR+ CD14-	DR	MR Egger	26.89451509	10	0.002706291
HLA DR on CD33br HLA DR+ CD14-	DR	Inverse variance weighted	28.92405415	11	0.002332363
CD80 on CD62L+ plasmacytoid DC	DR	MR Egger	12.05347706	9	0.21031129
CD80 on CD62L+ plasmacytoid DC	DR	Inverse variance weighted	12.64281383	10	0.24433363
CD80 on myeloid DC	DR	MR Egger	13.23045436	13	0.430172525
CD80 on myeloid DC	DR	Inverse variance weighted	13.31746917	14	0.501692218
CD28+ CD45RA- CD8dim AC	DR	MR Egger	10.62901657	14	0.714902326
CD28+ CD45RA- CD8dim AC	DR	Inverse variance weighted	11.89584092	15	0.686895401
CD33- HLA DR- AC	DR	MR Egger	3.6932846	9	0.930416234
CD33- HLA DR- AC	DR	Inverse variance weighted	3.710194195	10	0.959475108

DR, diabetic retinopathy.
